# Barriers for introducing HIV testing among tuberculosis patients in Jogjakarta, Indonesia: a qualitative study

**DOI:** 10.1186/1471-2458-8-385

**Published:** 2008-11-12

**Authors:** Yodi Mahendradhata, Riris Andono Ahmad, Pierre Lefèvre, Marleen Boelaert, Patrick Van der Stuyft

**Affiliations:** 1 Department of Public Health, Faculty of Medicine, Gadjah Mada University, Sekip Utara 55281, Jogjakarta, Indonesia; 2 Epidemiology and Disease Control Unit, Public Health Department, Institute of Tropical Medicine, Nationalestraat 155, Antwerp, Belgium

## Abstract

**Background:**

HIV and HIV-TB co-infection are slowly increasing in Indonesia. WHO recommends HIV testing among TB patients as a key response to the dual HIV-TB epidemic. Concerns over potential negative impacts to TB control and lack of operational clarity have hindered progress. We investigated the barriers and opportunities for introducing HIV testing perceived by TB patients and providers in Jogjakarta, Indonesia.

**Methods:**

We offered Voluntary Counselling and Testing (VCT) to TB patients in parallel to a HIV prevalence survey. We conducted in-depth interviews with 33 TB patients, 3 specialist physicians and 3 disease control managers. We also conducted 4 Focus Group Discussions (FGDs) with nurses. All interviews and FGDs were recorded and data analysis was supported by the QSR N6^® ^software.

**Results:**

Patients' and providers' knowledge regarding HIV was poor. The main barriers perceived by patients were: burden for accessing VCT and fear of knowing the test results. Stigma caused concerns among providers, but did not play much role in patients' attitude towards VCT. The main barriers perceived by providers were communication, patients feeling offended, stigmatization and additional burden.

**Conclusion:**

Introduction of HIV testing among TB patients in Indonesia should be accompanied by patient and provider education as well as providing conditions for effective communication.

## Introduction

Indonesia is critical to the global tuberculosis (TB) control efforts and increasingly important in the global HIV control efforts. The country ranks third in the world for TB burden [1]. The number of reported AIDS cases has increased by 15 fold in the past ten years [2]. The rapid increase of new HIV infections in Indonesia makes the epidemic one of the fastest growing in Asia, even though the aggregate national prevalence is as low as 0.16% [3]. By the end of 2007, there were 296 Voluntary Counselling and Testing (VCT) clinics throughout Indonesia, in addition to 153 hospitals which provide free antiretroviral treatment [3]. Patients with HIV-TB co-infection are appearing in hospitals and jails across several provinces and TB is a leading opportunistic infection among AIDS patients [4]. These trends suggest a potential of a dual HIV-TB epidemic, which many other developing countries, particularly in Sub-Saharan Africa are already facing.

WHO Interim Policy on HIV-TB recommends HIV testing among TB patients as an entry point for integrated HIV-TB care and surveillance [5]. However, scaling-up of this policy has been lagging [6]. Concerns over stigmatization which may generate TB patients unwillingness to use HIV associated services (with potential negative impact on TB case detection) and lack of detailed operational guidelines are among the important barriers [6,7].

Additionally, there is an ethical debate surrounding HIV testing among TB patients, particularly with regard to the unlinked anonymous testing method, in view of the improved prospects for HIV/AIDS treatment [8]. This led to linked confidential testing through an 'opt in' approach, which has been offered in Voluntary Counselling and Testing (VCT) centres [9]. More recently, WHO encouraged the adoption of provider-initiated linked confidential testing and counselling (PITC) [10]. In contrast to VCT, PITC is based on an 'opt out' approach in which the clinician initiates counselling when an individual is seeking medical care with signs or symptoms compatible with HIV infection [9].

Ultimately, decisions about how to implement HIV testing in TB patients, should be guided by an understanding of issues surrounding HIV testing among TB patients from the local stakeholders' perspectives [11]. Studies on groups other than TB patients suggest that knowledge, fear and access may constitute important barriers to HIV testing [12-14]. This study aimed to shed light on the issue through investigating the barriers for introducing HIV testing perceived by TB patients and providers in Jogjakarta, Indonesia.

## Method

### Study context

Jogjakarta province is located in the central part of Java island. It is divided into five districts, has 3.2 million inhabitants and covers an area of 3,185 square km. The province's primary care network consists of around 650 private practices and 117 public community health centres staffed with doctors, midwives and nurses. These first line services are backed up by 9 public hospitals and 24 private hospitals. The backbone of NTP's DOTS (Directly Observed Treatment, Short-course) programme in Jogjakarta province comprises a network of the 117 public health centres, 5 chest clinics and 18 public and private hospitals.

HIV prevalence among the general adult population in Jogjakarta province is 0.15–2.0% [15]. It is much higher among high-risk groups, e.g. sex workers [4.6 (3.6–6.4)%]; injecting drug users [39.3(29.0–52.7%)]. VCT services have been established in four hospitals and one NGO clinic. The standard procedure in these VCT services, in accordance to WHO guidelines for settings with HIV prevalence = 10% [16], requires three HIV tests (two rapid and one Enzyme Immunoassays test). Patients would have to return the next day to obtain all three test results. These VCT services are free of charge for all, including TB patients, through financial support from the Global Fund to fight AIDS, TB and Malaria.

### Study design

The study was conducted in parallel to a HIV prevalence survey among TB patients carried out between April and December 2006. The survey targeted TB patients attending all (88) public and private DOTS services in three out of five districts in the province. TB patients in participating health facilities were offered unlinked anonymous HIV testing for survey purpose and additionally free services of four hospital-based VCT centres. Nurses provided patients with standardized information on HIV and VCT services aided by a brochure which was subsequently given to the patient. If the patient expressed interest, nurses made an appointment with a VCT centre and provided an incentive to cover transport expenses to the centre. Out of 1269 TB patients whom were offered unlinked anonymous testing during the survey, 989 (77.9%) accepted [17]. The HIV prevalence was 1.9% (95% CI 1.6–2.2%) [17]. Out of these 989 patients, 133 (13.4%) expressed interest in VCT but only 52 (39.1%) subsequently attended VCT.

**Figure 1:**
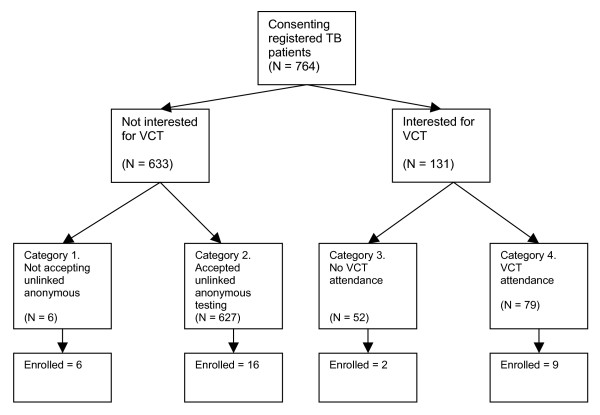
Patient flow.

The patients were asked whether they would be willing to be recruited for follow up in-depth interviews. We grouped the patients who accepted into four groups: (1) patients who refused unlinked anonymous testing and expressed no interest in VCT; (2) patients who accepted unlinked anonymous testing and expressed no interest in VCT; (3) patients who expressed interest, but did not attend VCT; and (4) patients who attended VCT. Among 1269 patients offered unlinked anonymous testing and VCT service during the parallel survey, 764 accepted to be interviewed. Figure 1 presents the distribution of these consenting patients by the 4 patient categories. We aimed to purposively sample eight patients within each group, keeping in mind the type of health facility attended and additionally age, gender, education and urban/rural residency. Appointments were made by nurses for the in-depth interviews of selected patients.

We interviewed 33 patients: 6 patients for group 1; 16 patients for group 2; 2 patients for group 3; and 9 patients for group 4. We faced difficulties recruiting patients for group 3 because the interview was perceived as a blaming attempt since they had received an incentive to cover transport to VCT, but had not attended. The large number of patients in group 2 was due to the need to increase the number of interviews to make up for the limited information collected from the first 8 respondents related to their very poor knowledge about HIV/AIDS. Patients were interviewed on the basis of an in-depth interview guide on why they were interested or not interested in VCT and probed for factors that hinder or support VCT uptake, e.g. knowledge, attitudes, information given by health providers regarding VCT.

Barriers preventing DOTS services providers to offer VCT services were also explored. We investigated nurses' perceptions through four Focus-Group Discussions (FGDs) sampling the different health facility types: (1) urban health centres; (2) rural health centres; (3) private hospitals; and (4) public hospitals and chest clinics. Within each group, we purposively selected nurses who were most involved in the offering HIV testing among TB patients and represented facilities with variation of patients' interest rate toward HIV testing. Each group consisted of eight to nine nurses. We finally carried out three in-depth interviews with all the specialist physicians providing DOTS services in public and private hospitals and with the three district disease control managers.

The in-depth interviews and FGDs were conducted by the first and second author.

### Data analysis

We recorded and fully transcribed all in-depth interviews and FGDs. Data analysis was supported using the QSR N6^® ^software (QSR International Pty. Ltd., Melbourne, Australia, 2002). The analysis was inductive which implies that categories of analysis were not imposed *a priori *on the data but are identified through the analysis process [18]. Transcripts imported into the software database were scrutinized to identify emerging and recurrent themes and a codebook was progressively established and structured. Text units were coded systematically. Coding frequency permitted to identify key issues and trends regarding perceptions of patients and providers about barriers to HIV testing.

### Ethical issues

We safeguarded confidentiality of patients' serostatus by unlinking HIV test results from our patients' identities. Informed consent was obtained from all respondents prior to data collection. All collected data were kept anonymous. Ethical approval for the qualitative data collection and the HIV-TB prevalence survey was given by the ethical review committee of the Faculty of Medicine, Gadjah Mada University, Indonesia.

## Results

### Patients' characteristics

Table 1 presents the characteristics of the interviewed patients' for the four categories. There were slightly more males then females among the patients. In general, they were predominantly aged between 20–40 years old, married, had secondary education and were offered VCT services by a public care provider. The groups' characteristics were in general similar with the exception of group 1 having slightly more old patients and group 4 having more patients attending public health facilities.

**Table 1:** Characteristics of enrolled TB patients

Patients' Characteristics	Patients' category*				Total N (%)
		
	Group 1 N (%)	Group 2 N (%)	Group 3 N (%)	Group 4 N (%)	
Gender					
Male	4 (66.7)	8 (50.0)	1 (50.0)	5 (55.6)	18 (54.5)
Female	2 (33.3)	8 (50.0)	1 (50.0)	4 (44.4)	15 (45.5)
					
Age group					
15–19 years old	0 (0.0)	0 (0.0)	1 (50.0)	1 (11.1)	2 (6.1)
20–29 years old	2 (33.3)	9 (56.3)	1 (50.0)	4 (44.4)	16 (48.5)
30–39 years old	0 (0.0)	8 (31.3)	0 (0.0)	1 (11.1)	6 (18.2)
40–49 years old	0 (0.0)	2 (12.5)	0 (0.0)	3 (33.3)	5 (15.2)
> 49 years old	4 (66.7)	0 (0.0)	0 (0.0)	0 (0.0)	4 (12.1)
					
Education					
Primary	1 (16.7)	2 (12.5)	0 (0.0)	2 (22.2)	5 (15.2)
Secondary	3 (50.0)	11 (68.8)	0 (0.0)	4 (44.4)	18 (54.5)
Tertiary	2 (33.3)	3 (18.8)	2 (100.0)	3 (33.3)	10 (30.3)
					
Married					
Yes	4 (66.7)	11 (68.8)	1 (50.0)	5 (55.6)	21 (63.6)
No	2 (33.3)	5 (31.3)	1 (50.0)	4 (44.4)	12 (36.4)
					
Health facility type					
Public	3 (50.0)	11 (68.8)	2 (100.0)	8 (88.9)	24 (72.7)
Private	3 (50.0)	5 (31.3)	0 (0.0)	1 (11.1)	9 (27.3)
					
TOTAL	6 (100.0)	16 (100.0)	2 (100.0)	9 (100.0)	33 (100.0

*Patients category:

• Group 1. Not accepting unlinked anonymous and not interested for VCT.

• Group 2. Accepted unlinked anonymous but not interested for VCT.

• Group 3. Accepted unlinked anonymous, expressed interest but did not attended VCT.

• Group 4. Accepted unlinked anonymous and attended VCT.

### Factors influencing patients' interests in VCT

Many of our respondents (22) were not interested to attend VCT regardless of gender, age, education and marital status. Most patients (24) had no negative feeling towards the HIV test offer, though some (9) clearly felt offended:

Frankly, that time I was offended. From the beginning, it was already explained that HIV is transmitted by this and that, not all drug users get it, also not all 'others' [risk groups] get it. And then all the sudden they offered me HIV test? *23-year-old, male, university student, attended VCT*

Knowledge of many respondents (11) on HIV was poor, ranging from those who had never heard of HIV to those who knew little. Patients with limited knowledge were less interested in VCT:

The problem is I don't even know what HF [HIV] is. Is it a new disease? I am just a lay person, so I don't know. It was my son who replied. [I told him] you should respond because you are the one who can answer. *52 year-old, male, employee, not interested in VCT*

Well what can I say? That HIV is not scary. It's just another disease. It can be cured. *29-year-old, female, employee, not interested in VCT*

Misconceptions regarding transmission of HIV/AIDS were common:

You can get infected through having a [sexual] relationship or through drugs or through smoking cigarettes, that's all I know. I heard it before from stories, you know, on TV. *26-year-old, male, unemployed, attended VCT*

I would imagine, that people who get infected by HIV are those who keep changing partners. If one doesn't change partners and does not use illegal drugs, then probably [he/she] can't get infected. *45-year-old, male, construction worker, attended VCT*

Table 2 summarizes the relations between main patients' perceptions and VCT interest. Many patients (16) did not report to perceive themselves at risk, or simply did not know enough to attribute risk (10):

**Table 2:** Patients' perceptions and interest for VCT

Patient's perception		Interested for VCT
At risk of being infected	Yes	majority
	No	minority
VCT entails benefits	Yes	roughly half
	No	small minority
HIV patients are stigmatized	Yes	roughly half
	No	minority
Fear of knowing test result	Yes	small minority
	No	vast majority
Access to VCT is a burden	Yes	minority
	No	vast minority

It's just for a test. It's not because one gets TB that one will get HIV. I've never done anything [wrong]. So I don't mind and I am also looking for a new experience. I am confident that the result will be non-reactive. No worries whatsoever. I am sure, Insya Allah [God's willing], as the doctor already know, that I won't get it. I imagine if one gets it. Oh my God! *37-year-old, male, employee, attended VCT*

I mean usually those who get HIV are those who like to go out at night, they like to...well, like commercial sex workers, they're like that, so they must get it. I never go out at night. I hardly leave my house. How can I get HIV? *29-year-old, female, employee, attended VCT*

A few patients (7) accepted that they could be at risk and were interested in VCT:

I've never done anything wrong [risky], or had a [sexual] relationship with someone with HIV. I've never received blood transfusion, never. I don't believe I can get HIV but, there's a possibility I get it because of TB, they say that can make you get infected easily. *45-year-old, male, construction worker, attended VCT*

No, I was already told [by the health worker] that from ...from the lungs it can lead to HIV. So I already knew beforehand. *24-year-old, female, self-employed, attended VCT*

Nearly half of the patients (16) perceived a certain benefit of HIV testing, regardless of whether they reported to perceive themselves at risk or not. Many of these (9) expressed interest towards VCT:

Well, to be able to know [whether I get] AIDS or ...HIV. I was not surprised [to be offered HIV testing]. I wanted to be examined to see if I had other diseases. *26-year-old, male, unemployed, attended VCT*

Some patients (10) perceived some stigmatization towards people living with HIV in the society. Others (8) did not perceive stigmatization, while the remaining participants (15) had no opinion. Most of those who perceived stigmatization (6) however were interested in VCT:

[They are] afraid to get infected, yes. Also afraid of ... what else...Well, it's a shameful and horrible disease. It's terrifying. So I would be afraid to be isolated, to be treated as someone infectious, as someone who has a pathetic disease. If I can, I will just avoid such disease. *29-year-old, female, attended VCT*

Well, the problem is AIDS is... Well, it is a shameful disease. I don't know... The problem is most people who get AIDS are those who do wrong things. People where I live, if they know, they will avoid you immediately. *17-year-old, female, student, attended VCT*

Some patients (5) feared knowing the HIV test result and were not interested in VCT, or initially expressed interest, but eventually changed their mind:

Why did it go that far? Saying HIV was like this and that. That made me scared. It's about psychology, I am sure I don't have HIV, but I am not mentally ready. It's enough that I got TB. If for instance I had to be tested for something like that [HIV], it could make things more complicated with so many problems...Oohhh! *23-year-old, male, student, not interested in VCT*

If they take my blood again, then they will test it, then if it turns out that I have that disease, it's like being struck on the head, it's a mental burden. What I am afraid of is that there is no cure yet, you die because of HIV. So if there's no treatment you will just die. *23-year-old male, student, initially expressed interest, but did not attend VCT*

A number of patients (8) also perceived burden for accessing and utilizing VCT. Most of these (6) were not interested in VCT.

The process would become too cumbersome. When I think about it, it will just make the process longer and complicated. My intention to seek treatment was just to get my coughs cured. *25-year-old, male, self-employed, not interested in VCT*

Well, at that time I thought, if they can do it at that moment, I wouldn't mind. I thought it would take too much time. [I asked] how I would know the result. [They said] if I wanted to know I have to go there. How can I manage the time? *51-year-old, male, employee, not interested in VCT*

### Nurses' perceptions

Table 3 depicts the distribution of main issues perceived by nurses across different type of health facilities. Most nurses considered their knowledge of HIV-TB insufficient:

**Table 3:** Nurses' perceptions of barriers to introduce HIV testing among TB patients*

Perception	Health centres		Hospitals and chest clinics	
	
	Rural	Urban	Public	Private
'Hard' patients	-	-	+	++
Additional burden	+/-	+/-	++	++
Patients offended	-	-	++	++
Stigmatization	+	+/-	+++	++
Lack of facility	+	+++	+/-	+
Communication difficulty	+/-	+/-	++	+++

* "+++" = critical; "++" = very important; "+" = "important"; "+/-" = less important; "-" = negligible

At the least, the lab technician, TB worker, nurse and doctor should know about the HIV issue comprehensively. Sometimes we go for training and bring home materials, but we don't really read them. There are patients who really need information on what is the relevance, goals. Yesterday there were two like that. At the end I had to read, I had to open the reference for them. The problem is we ourselves do not understand HIV comprehensively. *Female, nurse, rural health centre*

Nurses especially in the hospitals perceived that there are patients difficult to deal with, for instance skeptical highly educated patients. Nurses in hospitals also more frequently perceived offended patients as an issue:

Once we had a patient who was a high school teacher. We discussed how TB is the leading opportunistic infection for HIV. At the end it became confusing because the theory was not clear. At the end she refused. So how can we deal with patients who are highly educated? *Female, nurse, rural health centre*

Even though we have explained this and that...but in the end it doesn't seem to suffice. We really are not effective. *Female, nurse, public hospital*

The majority reacts negatively [to the offer]. Patients feel they have never done any wrongdoings. Patients feel they could not get it. Especially the VIPs [Very Important Person – Patients in first class wards]. All the VIPs refused. *Female, nurse, private hospital*

Lack of facilities was an issue perceived by nurses of all types of structures:

The room is still mixed [with other patients]. So, if possible, a separate room, which would be better to give patient education. It's inconvenient for us to do it when there are other patients around. *Male, nurse, public hospital*

We don't have a special room. Our place until now is semi-permanent, so mixed Maybe it wasn't convenient to offer the test to the patients under such condition. *Female, nurse, rural health centre*

Nurses at all facilities perceived some burden due to having to offer an HIV test, particularly with limited time available:

We don't have enough staff, for our lung clinic. It's just me and one assistant. If there are many patients we really don't have time, really too overwhelmed to offer [HIV testing]. We have more time in the morning. Those patients who accepted the offer are usually those who come in the morning. *Female, nurse, private hospital*

Nurses in hospitals particularly perceived difficulties in communication, mainly when it comes to patients who are 'hard' to deal with:

If they have detailed questions we have difficulty in explaining in details. We can handle general questions, but university students ask a lot of questions which are beyond our knowledge. *Female, nurse, private hospital*

Stigmatization of people living with HIV/AIDS within the community was perceived to be a barrier, particularly in hospitals:

They had fear, what if they turn out to be [HIV] positive? What would happen when they have to face the community. Some of them are community leaders. *Female, nurse, private hospital*

Strikingly, a few nurses' comments suggested that some nurses stigmatize people living with HIV:

We're also worried, what if nurses get it too? It will [then] become very risky for [HIV-negative] patients. We need to isolate them if we can identify them, but until now we don't know who is positive and who is not. Even if it's [just] gonorrhoea and somebody [staff] knows, everyone [staffs' behaviour] becomes different. *Female, nurse, public hospital*

### Perceptions of decision makers: specialists and disease control managers

Both specialists and disease control managers perceived patient-provider communication and stigmatization as important barriers to VCT uptake:

Yes, I've observed that some health workers really can't talk, they can't communicate. Really, it's not that they don't want to do it, but they simply don't have the capacity to do it. So we can't do anything, because they are all we got. *Female, disease control manager, urban district*

What I liked about the programme [introducing HIV testing among TB patients] was that the TB patients got more attention. There was a demand to the health worker to be able to communicate better. We basically have nurses and doctors who can communicate well, but the majority have limited communication skills and it's not just a matter of education, it's also about personality. *Male, disease control manager, rural district*

Specialists seemed to be more optimistic, giving more emphasis on the managerial challenges than on the operational:

The most important thing is that this is integrated at the top level. If this is still under two different national programmes then it will be difficult for policy making. If it's integrated at the top level, [we] at the frontline just have to implement. But if at the top there are still two heads, what can we do? It's a sensitive issue, but that's the reality. *Male, senior lung specialist, public chest clinic and private hospital*

The management system needs to be repaired. If we're integrating TB and HIV, the management becomes more [crucial]. Especially that we're involving two different national programmes together. The financing, the organization... *Male, junior lung specialist, teaching hospital and private hospital*

They also perceived much less additional burden:

I don't feel any [significant] additional burden. As far as I've observed, care delivery was not disrupted. Of course there were some additional things [tasks], but not so much. *Male, senior lung specialist, public chest clinic and private hospital*

However, specialists strongly perceived lack of knowledge on HIV to be a major hindrance to introduce testing, including among colleagues:

Even in this hospital, other specialists don't really know [about HIV]. Internal medicine and dermatovenereology [specialists] know quite a bit, but others still ask a lot of questions. They only know it superficially. *Male, internist, private and teaching hospital*

But both district control managers and specialists were not concerned with potential harms to the TB control programme's performance:

"No, I am not worried, the patients were not obliged to be tested ... and I've observed no reduction of case reporting so far. Our patients were not running away". *Female, disease control manager, urban district*

## Discussion

Previous studies examining the motivations and deterrents to HIV testing have been carried out mainly among groups other than TB patients, i.e.: pregnant women [14,19]; drug users [12,20]; poor population [21]; and multiple risk groups [13,22]. Our study contributes to the evolving body of evidence on specific factors that influence introduction of HIV testing among TB patients. This study is limited by qualitative research boundaries. Issues perceived by patients and providers were identified. Although trends emerge, the respective influence of each issue was not quantified. This could be documented through a quantitative survey building on our findings, which points out the key issues to be taken into account. We have focused on contrasts between patients who expressed and did not express interest for VCT because only two patients who expressed interest but did not attend could be interviewed (group 3) and because we interviewed more patients who did not express interest but accepted unlinked anonymous (group 2). This means our findings can be interpreted in terms of VCT uptake rather than interest. Although our findings are context bound, generalization can be considered to other provinces in Indonesia with similar socio-economic, HIV-TB epidemiology and health system characteristics. Some specific findings may hold in similar settings in other countries.

### Knowledge

Knowledge of TB patients on HIV and its transmission was strikingly poor with considerable misconceptions, particularly regarding transmission routes. Pregnant women in Hong Kong and China reportedly also had inadequate knowledge regarding HIV transmission [14,23]. Poor knowledge of HIV among the general population in the US and pregnant women in Hong Kong is associated with poor uptake of HIV testing [14,22]. In addition, our findings suggest that knowledge of providers regarding HIV and HIV-TB is also insufficient. A similar lack of knowledge particularly regarding HIV testing among physicians was documented in India [24,25]. The need for professional education to precede VCT programmes has also been further affirmed by a study among health workers in China [23].

### Stigmatization

Our data suggests that stigmatization of HIV is present in the Indonesian society. HIV/AIDS has been one of the most stigmatized diseases of the last 20 years [26]. HIV-associated stigma has remained a barrier to testing among pregnant women in China [23]. Perceived stigmatization among mineworkers in South Africa and urban inhabitants in Mali reportedly also deterred them from HIV testing [27,28]. Stigmatization was also considered to be an important barrier to HIV testing by nurses in our study. Our findings further show that there are even nurses who also stigmatize HIV patients. This is similar to the findings from China in which 30% of health workers would not treat HIV patients [23]. However, our data suggests that stigmatization did not play much role on patients VCT interest. Most likely this is because HIV/AIDS in our setting is not yet a widespread disease with high visibility. Other factors outweigh stigmatization when it comes to interest in VCT, e.g. a clear indication of the risk for HIV infection, as effectively communicated by the care provider, coupled with patients' concerns for their personal well-being.

### Perceived benefit and risk

Perceived benefit and risk showed considerable influence on VCT interest among our TB patients. Mineworkers in South Africa perceive HIV testing to be more acceptable if antiretroviral therapy (ARVs) become more available [27]. Rates of HIV testing tend to increase as perceived benefits increase. However, the most worrying HIV testing barrier is that people do not perceive themselves at risk [29]. The main stated reason for refusal of HIV screening among TB patients in Tamilnadu, India was 'no risk behaviour' [30]. Some drug users in the US indeed did not test for HIV as they had not perceived themselves at risk [12]. Perception of not being at risk persists as a barrier to testing in the US, despite self-report of high-risk behaviors [13]. We likewise encountered a similar tendency among our TB patients.

### Fear of knowing the test result

Our findings indicate that fear of knowing test result plays a role in VCT interest. Such fear has also been documented as a barrier among risk populations in the US [13]. A survey among Indonesian drug users in Bali province documented that the most important reason for avoiding HIV testing (55% respondents) was fear of positive results [20]. A qualitative study carried out more recently in the same risk population affirmed the importance of fear of knowing the test result as a barrier [31].

### Perceived burden for utilizing VCT

In addition to transportation, our patients still had to spend considerable time waiting for the counselor to see them, undergo the counseling process, have their blood taken, return home and come back again the next day for the result. The length of the process, linked to the perception of not being at risk, was enough to deter most patients. Our TB patients were offered transport incentives, but this did not help much. Other studies have documented similar observations. Some Indonesian drug users refused testing because of the long wait and complicated procedures [20]. Accessibility of the VCT centres has been shown to motivate TB patients in India to undergo testing for HIV [30]. Drug users in the US decided to test because the site was immediately available and they need not travel far [12].

### Communication

A main barrier from the providers' side was related to communication. Providers attributed this problem to difficulties to communicate on HIV issues, lack of time and adequate facilities. The disease control managers stated that health workers hardly communicate with patients and that some health workers did not have proper communication skills. Patient-provider communication around HIV in resource-constrained setting seemingly falls short of best-practice standard [25].

Our findings additionally revealed that communication was influenced by characteristics of the patient, provider and healthcare facility conditions. The worst case scenario occurs when a skeptical highly educated patient comes into contact with a nurse worker with poor communication skills in an overburdened hospital. This highlights the need for creating the material conditions in the health services which make it easier for health workers to interact with patients. Indonesia's health services were designed to cope with acute diseases and the existing service delivery model is clearly not conducive to effective VCT. HIV/AIDS is a complex chronic condition requiring long-term involvement, patient-centered approaches and patient-provider communication starting from the point of HIV testing offer.

The magnitude of communication problems identified in this study was not evenly distributed across health facility types and was more prominent in hospitals especially private. These hospitals are overloaded with patients. They also see more patients who are challenging to deal with. All of these issues have to be managed under conditions of limited time, staff and facilities.

## Conclusion

TB patients evidently experienced multiple barriers that can deter them for HIV testing. The study highlighted that patients' and providers' knowledge regarding HIV was inadequate in our setting. The main barriers to HIV testing identified were: fear, burden to access VCT and communication problems. Stigma exists in society and caused concerns among providers, but did not seem to play much role in patients' interest in VCT.

If the Ministry of Health intends to move forward with linked confidential HIV testing among TB patients through VCT, provider's and patient's knowledge need to be improved simultaneously, the general healthcare system strengthened by providing the necessary conditions for effective communication and patient-provider interaction and offering VCT at potential DOTS services that can provide results on the same day. The potential acceptability of the alternative PITC model would be worth to explore further. However, it would clearly require even more demanding pre-conditions and thus should be reserved for settings with more advanced HIV epidemic. In any case, efforts to understand and overcome specific local barriers must accompany efforts to introduce HIV testing among TB patients.

## Competing interests

The authors declare that they have no competing interests.

## Authors' contributions

YM, RA, PL, MB and PVDS made substantial contributions to conception and design. YM and RA collected the data. YM, RA, PL, MB and PVDS made substantial contribution to analysis and interpretation of data. YM and PL have been involved in drafting the manuscript. YM, RA, PL, MB and PVDS have contributed to revising the manuscript critically for important intellectual content and have given final approval of the version to be published.

## Pre-publication history

The pre-publication history for this paper can be accessed here:


